# Microbial insights into the survival adaptation of the endemic orchid *Corybas fanjingshanensis* in a “sky island” habitat

**DOI:** 10.1128/spectrum.03719-25

**Published:** 2026-10-06

**Authors:** Huakai Zou, Jianghong Yu, Haibo Li, Jian Xu, Mingtai An, Li Tian, Yuhang Ma

**Affiliations:** 1 College of Forestry, Guizhou University 71206, Guiyang, Guizhou, China; 2 Key Laboratory of Plant Resource Conservation and Germplasm Innovation in Mountainous Region (Ministry of Education), College of Life Sciences, Institute of Agro-bioengineering, Guizhou University 71206, Guiyang, Guizhou, China; 3 Guizhou Fanjingshan Forest Ecosystem Observation and Research Station, Fanjingshan National Nature Reserve, Tongren, Guizhou, China; 4 Guizhou Botanical Garden 439463, Guiyang, Guizhou, China; Connecticut Agricultural Experiment Station, New Haven, Connecticut, USA

**Keywords:** sky island, narrowly endemic species, survival adaptation, Orchidaceae, microbiome, conservation biology

## Abstract

Understanding the microbiological basis of species adaptation is crucial for effective conservation. This study investigates the endangered, narrow-endemic orchid *Corybas fanjingshanensis* and its symbiotic moss substrate in a “sky island” habitat, using high-throughput sequencing to analyze microbial communities and their potential roles in host survival. Results reveal significant divergence between endophytic and environmental microbiomes. Endophytic bacterial diversity was lower, but dominance was higher than in the moss substrate. We identified 65 core bacterial and 18 core fungal genera, predominantly enriched in nutrient acquisition and stress tolerance functions. Five key bacterial taxa, including *Vicinamibacteraceae* and *Mucilaginibacter*, were highlighted as potential conservation targets. The *C. fanjingshanensis–moss* symbiotic network exhibited strong positive interactions and high modularity, forming tight functional clusters likely enhancing environmental stress resistance. While bacterial functional genes remained stable across communities, fungal nutritional strategies were influenced by environmental heterogeneity. We propose conservation measures, including microbial transplantation to rebuild host-microbe symbioses, selective amplification of core taxa, and a holistic framework integrating habitat integrity with long-term microbial monitoring. This study offers insights into the survival strategies of narrow-endemic plants and advances microbiome-based orchid conservation, shifting the paradigm from habitat simulation to microbial network restoration.

**IMPORTANCE:**

Using *Corybas fanjingshanensis*, a critically endangered species with an extremely limited distribution, as the research subject, high-throughput sequencing technology was employed to investigate its survival adaptations from a microbial perspective within the unique “sky island” habitat. The findings provide a reference for microbiome-based conservation of endangered narrow-endemic species and offer insights into plant habitat reconstruction.

## INTRODUCTION

Under the background of global climate change and intensified human activities, narrowly distributed plant species are highly sensitive to environmental disturbances due to their restricted ranges, specialized ecological niches, and small population sizes, which place them at high risk of extinction. Consequently, understanding their mechanisms of survival and adaptation has become a central focus in conservation biology and ecology (1–3). Among the various strategies contributing to species adaptability, symbiotic associations between plants and microorganisms are increasingly recognized as an intrinsic driving force (4, 5). Plants are not isolated entities but function as holobionts together with their symbiotic microbiota. These microorganisms act as extensions of the plant genome, enhancing metabolic and physiological capacities (6) and thereby improving resistance to both abiotic and biotic stresses (7, 8). Under nutrient-limited conditions, arbuscular mycorrhizal fungi expand the absorptive range of plant roots through their hyphal networks, facilitating efficient uptake of phosphorus, nitrogen, and water (9–11). Similarly, plant growth-promoting rhizobacteria can mobilize soil nutrients through phosphate solubilization, potassium release, and nitrogen fixation, thereby improving plant nutrition (12). In response to abiotic stresses, such as low temperature, drought, and salinity, specific microorganisms synthesize osmolytes and antioxidant enzymes, or modulate plant endogenous hormone signaling pathways, to maintain osmotic balance and scavenge reactive oxygen species, thus mitigating cellular damage under stress (13, 14). Moreover, within microbial communities, intricate positive and negative interactions form complex and stable co-occurrence networks that promote functional complementarity and resource sharing, enhancing the robustness of the holobiont system and maintaining its functional stability under environmental fluctuations (15). Therefore, elucidating the functional characteristics and co-occurrence network structures of plant-associated microbiomes is a key step toward understanding the mechanisms underlying plant adaptation to environmental changes.

Orchids are among the plant groups most closely associated with microorganisms, and microbial interactions profoundly influence their entire life cycle (16). Orchid seeds are minute and lack endosperm; therefore, their germination and seedling establishment usually depend on specific mycorrhizal fungi, which can provide the host with carbon sources, mineral nutrients, and water during early developmental stages (17–19). In addition to mycorrhizal fungi, bacteria colonizing the orchid rhizosphere, root tissues, and other plant tissues may also act as plant growth-promoting bacteria or mycorrhization-helper bacteria, thereby contributing to host nutrient acquisition, stress tolerance, pathogen suppression, and microbial colonization (20–23). Therefore, the long-term persistence of many terrestrial orchids depends not only on aboveground habitat conditions but also on the availability of suitable belowground or habitat-associated microbial reservoirs. However, existing studies have mostly focused on orchid mycorrhizal fungi or rhizosphere microorganisms in terrestrial habitats, whereas the relationships among orchid endophytes, habitat-associated microorganisms, and host adaptation in high-elevation, isolated “sky island” ecosystems remain poorly understood.

“Sky islands” refer to high-altitude mountain habitats isolated by heterogeneous lowland environments, characterized by distinct climatic conditions and unique biological communities (24). Plants inhabiting these environments are subject to dual selective pressures of geographic isolation and extreme environmental conditions. On the one hand, geographic isolation restricts gene flow among populations, increasing the risks of genetic drift and inbreeding depression, thereby reducing their genetic adaptability (25). On the other hand, persistent low temperatures, intense ultraviolet radiation, short growing seasons, and nutrient-poor soils at high elevations further constrain plant growth and development (26, 27). Under such circumstances, co-evolved microbial communities may play a compensatory role by enhancing the host’s adaptive capacity through collective metabolic functions and complex species interaction networks, providing essential support for plant survival in these extreme habitats. However, the patterns of microbial community assembly, functional adaptation, and their roles in host stress resistance within “sky island” environments remain largely unexplored.

*Corybas fanjingshanensis* is a narrowly distributed orchid species that occurs only in the alpine mossy dwarf forests on the summit of Fanjingshan, the main peak of the Wuling Mountains, and represents a typical “sky island” species. It mainly grows in moist, high-density moss communities rather than directly in exposed mineral soil. Therefore, moss habitats may serve as microbial reservoirs analogous to soil microhabitats. However, *C. fanjingshanensis* also faces multiple constraints, including a small and isolated population size, limited gene flow, low temperatures at high elevations, and relatively nutrient-poor environmental conditions that restrict its growth and development. Meanwhile, intensified global climate change may further increase the instability of alpine “sky island” habitats (28), readily affecting the growth of moss communities and disrupting the microecosystem on which *C. fanjingshanensis* depends. Therefore, *C. fanjingshanensis* may face a substantial risk of population decline, making the development of targeted conservation measures urgent. In this context, endophytes may represent a key biological factor contributing to the adaptation of *C. fanjingshanensis* to environmental stress.

Therefore, this study focused on *C. fanjingshanensis*, integrating high-throughput sequencing with multidimensional statistical analyses to systematically characterize the endophytic and environmental microbial communities and their interrelationships. Specifically, we addressed the following objectives: (i) to examine the composition and differentiation of endophytic and habitat microbial communities and explore the selective processes shaping microbial community assembly under the “sky island” context; (ii) to analyze the microbial co-occurrence network structure and the functional potential of shared core taxa within the *C. fanjingshanensis*–moss symbiotic system, revealing microbial strategies underlying their coordinated adaptation to alpine environments; and (iii) to propose targeted conservation measures based on microbial regulatory insights. This study provides a reference for understanding plant–microbe co-adaptation to environmental stress and the mechanisms maintaining plant diversity in alpine ecosystems under the “sky island” framework and offers a scientific basis for developing microecological conservation and microbiome-assisted restoration strategies for narrowly distributed species.

## MATERIALS AND METHODS

### Study area

**Fig 1:**
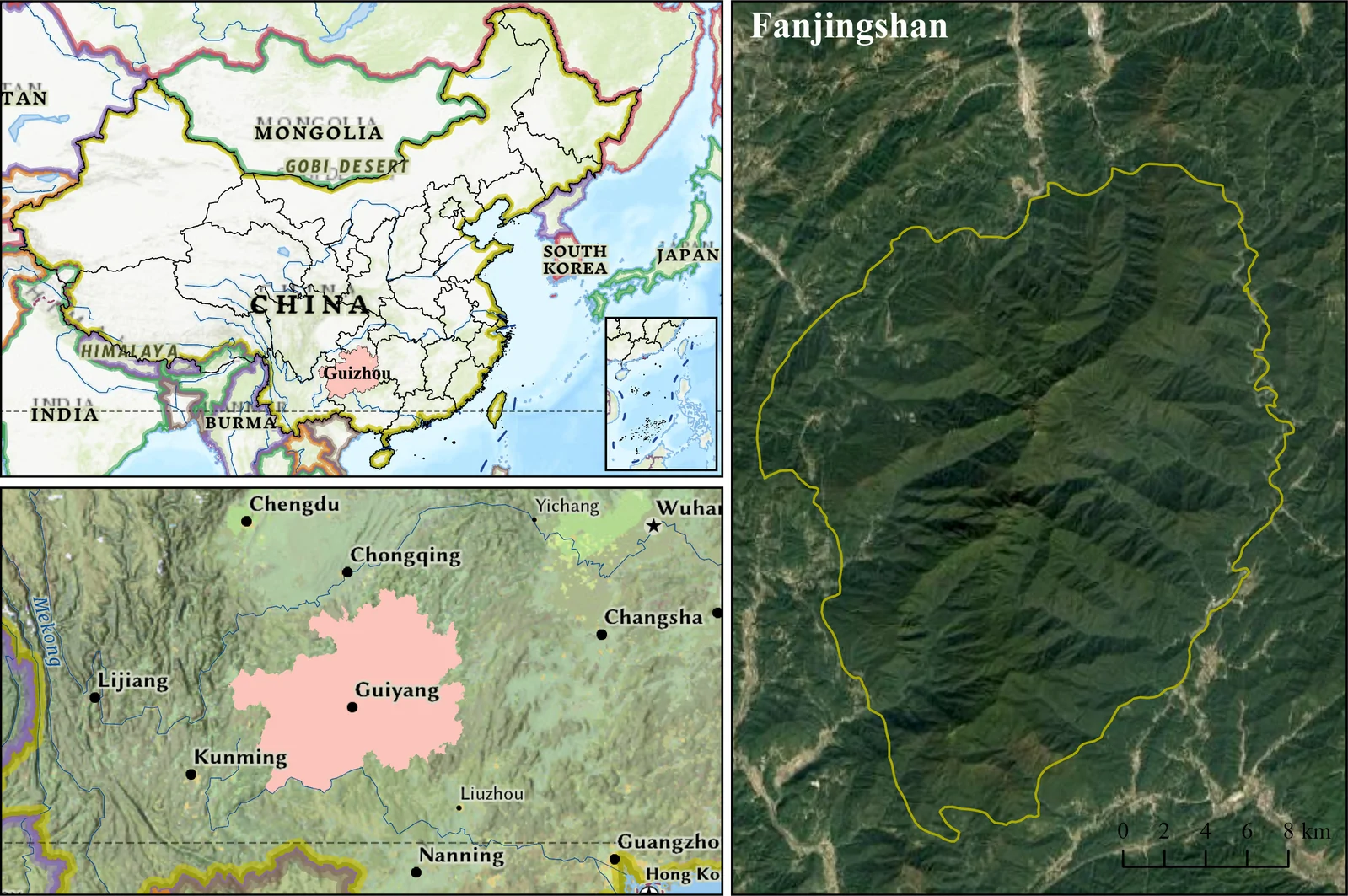
Map of the study area.

The Fanjingshan National Nature Reserve is located on the transitional slope between the Yungui Plateau and the western Hunan Hills in the second geomorphological step of China (108°45′55″–108°48′30″E, 27°49′50″–28°01′30″N), representing the main peak of the Wuling Mountains (Fig. 1). Its unique geological origin has formed a metamorphic rock massif uplifted from the surrounding karst landscape, which subsequently evolved into a terrestrial ecological island with an elevation of 2,572 m and a vertical relief of nearly 2,000 m (29). The region is located in the central subtropical monsoon zone of southwestern China, with an annual average temperature ranging from 6°C to 17°C along the elevational gradient, annual precipitation ranging from 1,100 to 2,600 mm, and an average relative humidity exceeding 80% (30). Owing to its massive topography and complex landform structure, the mountain exhibits pronounced spatial redistribution of hydrothermal conditions across elevations, resulting in a typical pattern of subtropical montane vegetation zonation. The temperature decreases by approximately 0.5°C–0.8°C for every 100-m increase in altitude (31, 32). These heterogeneous hydrothermal and topographic conditions support exceptionally rich and unique biodiversity. The vegetation coverage of Fanjingshan is significantly higher than that of the surrounding karst regions, making it an ecological “green island” rising above the karst “sea” (31). However, the ecological isolation of Fanjingshan differs from that of true oceanic islands (33) and from typical mid- to high-altitude terrestrial islands with incomplete isolation. Its distinctive ecological processes confer irreplaceable ecological and biodiversity value (34).

### Sample collection and processing

Extensive field investigations revealed that *C. fanjingshanensis* primarily grows within seven distinct moss communities (Table S1). Both *C. fanjingshanensis* individuals and their associated moss substrates were collected using 20 × 20 cm quadrats, with three replicates for each moss type. All samples were labeled and transported to the laboratory in ice boxes. The tissues of *C. fanjingshanensis* were first rinsed with sterile water to remove surface debris and then surface-sterilized under a laminar flow hood. Roots, stems, leaves, and tubers were carefully excised using sterilized scalpels, disinfected in 80% ethanol for 2 min, and subsequently rinsed with sterile distilled water. Each tissue type was placed into a sterile 2 mL centrifuge tube (approximately 2 g per sample). For the moss-layer samples, a small portion of each moss type was disinfected in 80% ethanol for 2 min, rinsed with sterile distilled water, and placed into sterile 5 mL centrifuge tubes (approximately 2 g per sample). To obtain the moss-water-layer samples, the remaining moss material was soaked in 1 L of sterile distilled water in an autoclaved beaker for 3 h. After removing the moss, the soaking water was first filtered through a 3 μm membrane to eliminate large particles and subsequently through a 0.2 μm membrane to collect microorganisms. The resulting filter membranes were air-dried, cut into small pieces with sterile scissors, and transferred into sterile 5 mL centrifuge tubes. All processed samples were stored at −80°C until further analysis. In total, 126 samples were obtained (seven moss types × three replicates × six sample types: moss, moss water, root, stem, leaf, and tuber) and subjected to Illumina MiSeq high-throughput sequencing, performed by GenePioneer Co., Ltd. (Nanjing, China). Furthermore, for ease of analysis, the tissue samples of *C. fanjingshanensis*, moss-layer samples, and moss-water-layer samples are hereafter abbreviated as CF, M, and MW, respectively.

### Microbial DNA extraction, amplification, and sequencing

Genomic DNA was extracted from the microbial communities using a Universal Genomic DNA Extraction Kit following the manufacturer’s instructions. DNA concentration was quantified with a Qubit 3.0 Fluorometer. Target regions were amplified by PCR using barcoded primers: the V3-V4 region of the bacterial 16S rRNA gene with primers 341F/805R (35, 36), and the ITS1 region of the fungal ITS gene with primers ITS1F/ITS2R (37, 38). Each 30 μL PCR reaction contained 15 μL of 2× Phanta Max Master Mix, 1 μL of each primer (10 μM), and 20 ng of template DNA. The amplification program consisted of an initial denaturation at 95°C for 5 min, followed by 30 cycles of denaturation at 95°C for 30 s, annealing at 55°C for 30 s, and extension at 72°C for 45 s, with a final extension at 72°C for 5 min. PCR products were verified by 2% agarose gel electrophoresis, purified using an AxyPrep DNA Gel Extraction Kit, and quantified. Equal molar amounts of purified PCR products (20 barcoded samples per pool) were combined to construct paired-end libraries according to the standard Illumina protocol. High-throughput sequencing was performed on the Illumina NovaSeq 6000 platform (GenePioneer Co., Ltd., Nanjing, China) with a 2 × 250 bp paired-end configuration.

### Data and statistical analysis

Raw sequencing data were processed and quality-controlled using the QIIME2 pipeline (v2021.11) (39). Denoising and chimera removal were performed with DADA2 (40) to generate amplicon sequence variants (ASVs). Taxonomic classification was conducted using a pre-trained Naïve Bayes classifier: bacterial ASVs were aligned against the SILVA 138 database (41), and fungal ASVs against the UNITE 8.0 database (42). Alpha diversity analysis, Venn diagrams, analysis of similarities (ANOSIM), linear discriminant analysis effect size (LEfSe), and heatmap visualizations were conducted in the R environment (v4.4.2). Principal coordinates analysis (PCoA) based on Bray–Curtis distances, microbial co-occurrence network construction, and module hub taxa identification were performed on the GenePioneer Cloud Platform (Nanjing GenePioneer Biotechnologies, https://www.genepioneer.cn/). Functional prediction of bacterial communities was performed using PICRUSt2 (43) with reference to the KEGG database (44), while fungal trophic modes were inferred using FUNGuild (v1.1) (45).

## RESULTS

### ASV clustering analysis of samples

**Fig 2:**
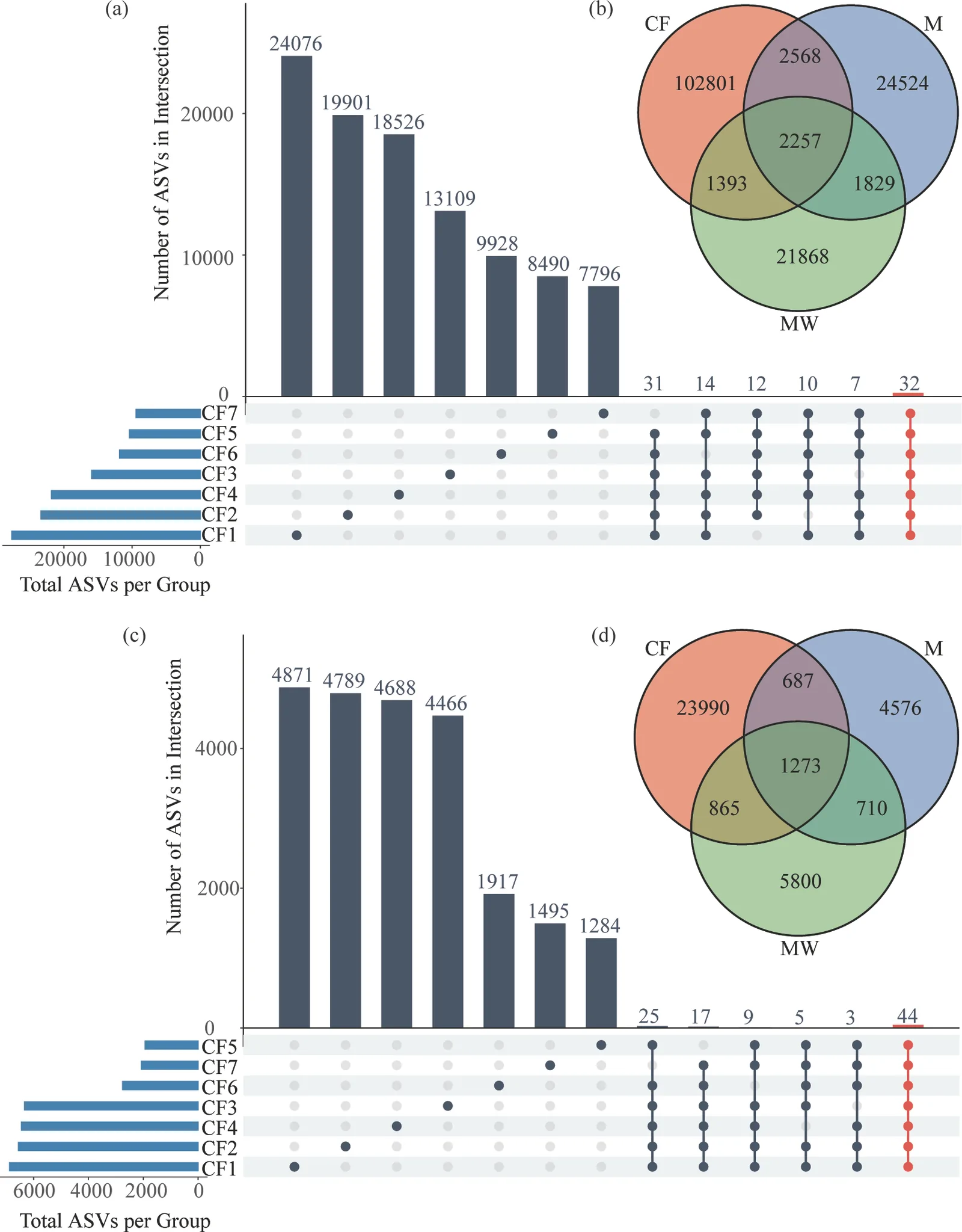
Venn analysis of bacterial and fungal amplicon sequencing variants. (**a**) Bacterial ASVs in *C. fanjingshanensis.* (**b**) Bacterial ASVs in *C. fanjingshanensis,* moss layers, and moss-water layers. (**c**) Fungal ASVs in *C. fanjingshanensis*. (**d**) Fungal ASVs in *C. fanjingshanensis*, moss layers, and moss-water layers. CF, *C. fanjingshanensis*; M, different moss layers; and MW, different moss-water layers.

A total of 10,865,252 raw bacterial reads and 12,927,316 raw fungal reads were generated by high-throughput sequencing. After quality control procedures, including trimming, filtering, and chimera elimination, 9,842,480 clean bacterial reads and 12,195,088 clean fungal reads were obtained. The number of valid sequences in CF, M, and MW compartments was 6,474,047, 1,726,055, and 1,642,378 for bacteria and 8,564,843, 1,767,726, and 1,862,519 for fungi. Further analysis revealed that the unique endophytes of CF were highly abundant across different sampling sites, whereas the shared endophytes were extremely scarce (Fig. 2a and c). Second, the richness of unique endophytic bacteria (102,801) and fungi (23,990) in CF was significantly higher than that in M (bacteria, 24,524; fungi, 4,576) and MW (bacteria, 21,868; fungi, 5,800). Furthermore, the number of ASVs shared among all three groups, as well as those shared between any two groups, was significantly lower than the number of unique ASVs (Fig. 2b and d). These results indicate that the endophytic microbiome of *C. fanjingshanensis* not only exhibits significant variation across different microhabitats (sampling sites) but also that the endophytic environment exerts a strong selective filtering effect on environmental microorganisms.

### Microbial diversity and community structure

**Fig 3:**
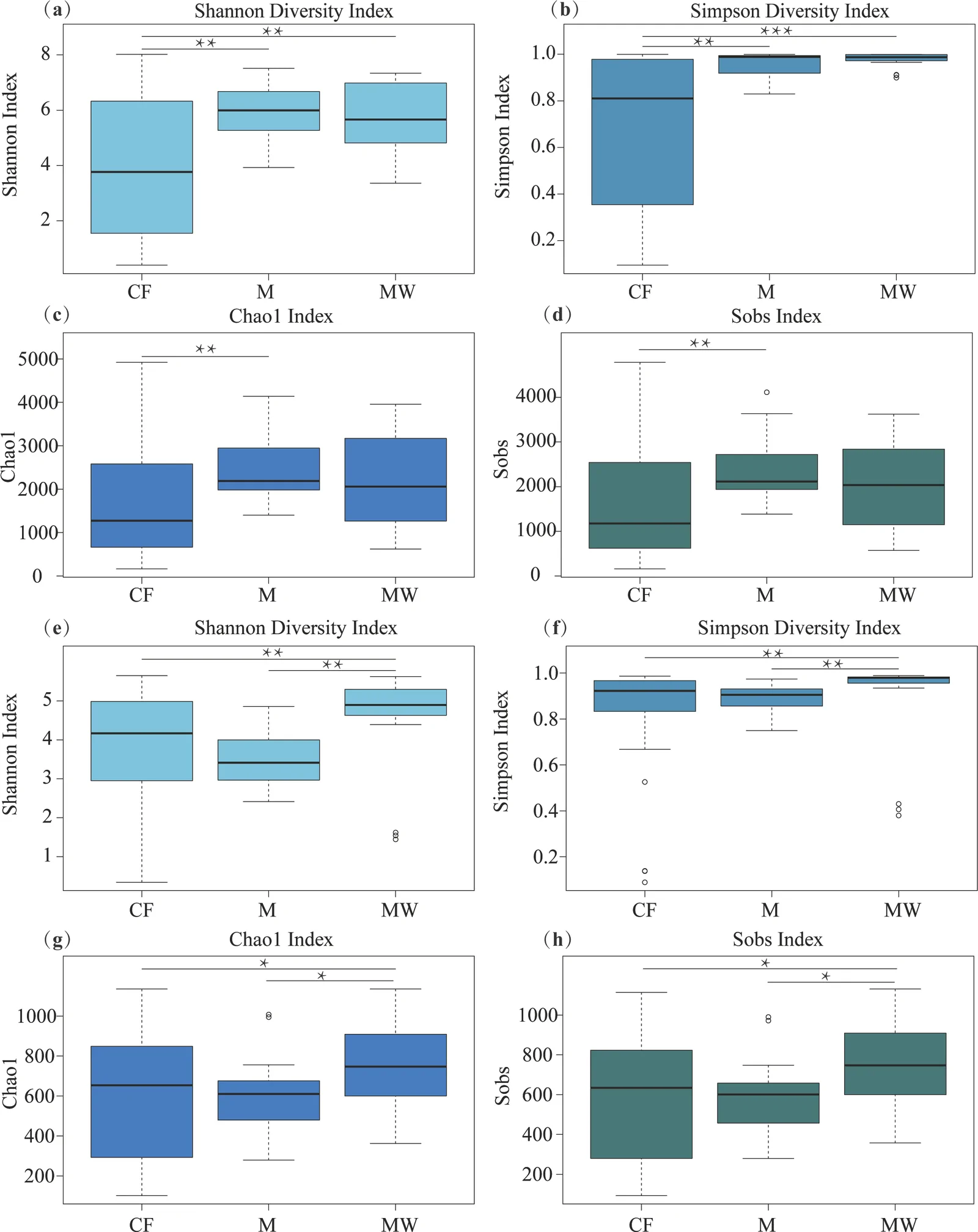
Alpha diversity analysis of microorganisms in *C. fanjingshanensis*, moss layers, and moss-water layers. (**a**) Bacterial Shannon diversity index. (**b**) Bacterial Simpson diversity index. (**c**) Bacterial Chao 1 index. (**d**) Bacterial Sobs index. (**e**) Fungal Shannon diversity index. (**f**) Fungal Simpson diversity index. (**g**) Fungal Chao 1 index. (**h**) Fungal Sobs index. CF, *C. fanjingshanensis*; M, different moss layers; and MW, different moss-water layers. *, *P* < 0.05; **, *P* < 0.01; ***, *P* < 0.001; and ****, *P* < 0.0001.

At the ASV level, alpha diversity analyses were conducted for microbial communities in *C. fanjingshanensis*, moss layer, and moss-water-layer samples. For bacterial communities, Shannon indices indicated that CF samples had significantly lower diversity than M and MW samples (Fig. 3a), while Simpson indices showed significantly higher dominance in CF communities (Fig. 3b), suggesting that the community structure is dominated by specific dominant taxa. In addition, both Chao1 and Sobs indices showed lower species richness in CF samples compared with M and MW samples (Fig. 3c and d). This may be related to the relatively closed endophytic environment of CF, which limits the influx of exogenous microorganisms, whereas M and MW, as intermediates connecting vegetation and the external environment, experience more frequent environmental contact and can harbor more bacterial species. Regarding fungal communities, MW samples had significantly higher Shannon, Chao1, and Sobs indices than CF and M samples (Fig. 3e, g, and h), while Simpson indices showed lower dominance in MW samples (Fig. 3f), indicating a more even distribution and more diverse composition of fungal communities. This may result from the exchange of substances and microorganisms between the moss-water layer and external water, allowing access to richer nutrients and ecological niches that support the growth and reproduction of more fungal species. Overall, endophytic bacterial and fungal communities in CF form structures dominated by specific taxa within a relatively stable and isolated endophytic environment, whereas moss layer and moss-water-layer communities, especially MW, exhibit higher diversity and richness due to more open habitat conditions and frequent interactions with the external environment.

**Fig 4:**
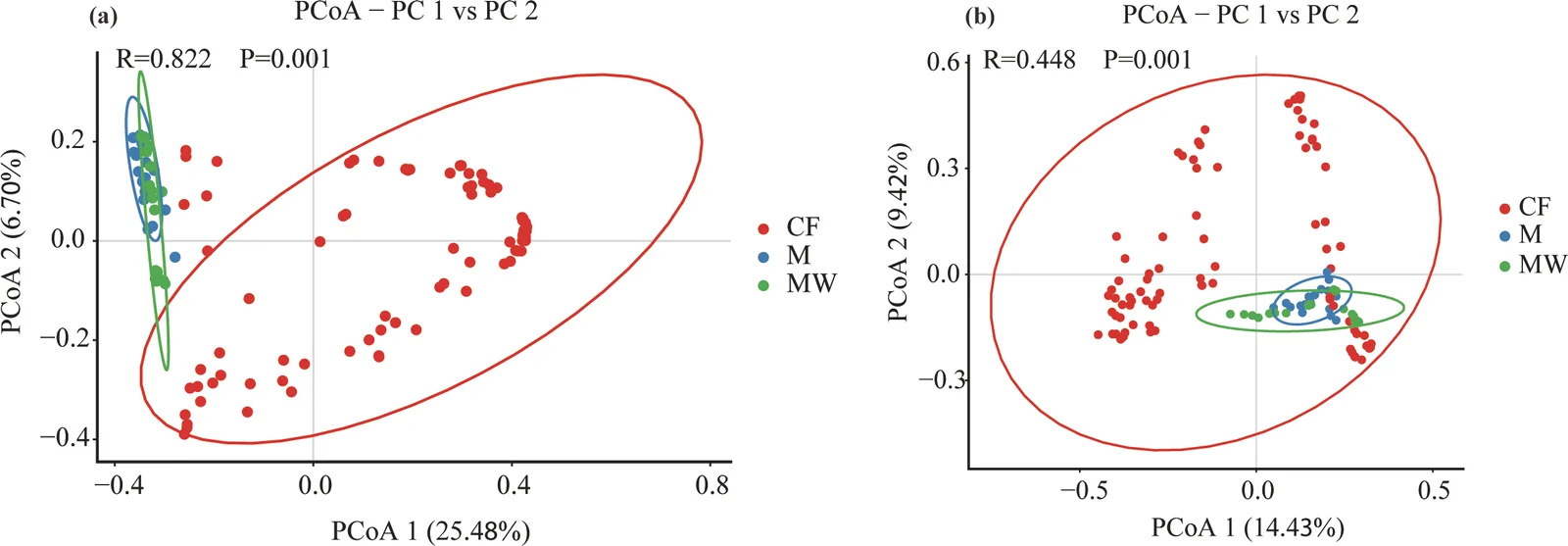
Principal coordinates analysis of microbial communities of *C. fanjingshanensis*, moss layers, and moss-water layers using Bray-Curtis distance. (**a**) PCoA of bacterial communities. (**b**) PCoA of fungal communities. *, *P* < 0.05; **, *P* < 0.01; and ***, *P* < 0.001. CF, *C. fanjingshanensis*; M, different moss layers; and MW, different moss-water layers.

To assess overall differences in microbial community structure among samples, PCoA was performed based on the Bray-Curtis distance matrix to evaluate β-diversity. The results showed that the first two axes of bacterial communities (PC1 and PC2) explained 25.48% and 6.70% of the variance, respectively (Fig. 4a), while for fungal communities, PC1 and PC2 explained 14.43% and 9.42% of the variance, respectively (Fig. 4b). CF, M, and MW samples were dispersed in the coordinate space, and no obvious clustering was observed within groups, indicating significant differentiation in microbial community composition among the three sample types. Further validation by ANOSIM confirmed that between-group differences were significantly greater than within-group differences (*P* < 0.001), supporting the reliability of the groupings.

### Microbial community composition and differences

**Fig 5:**
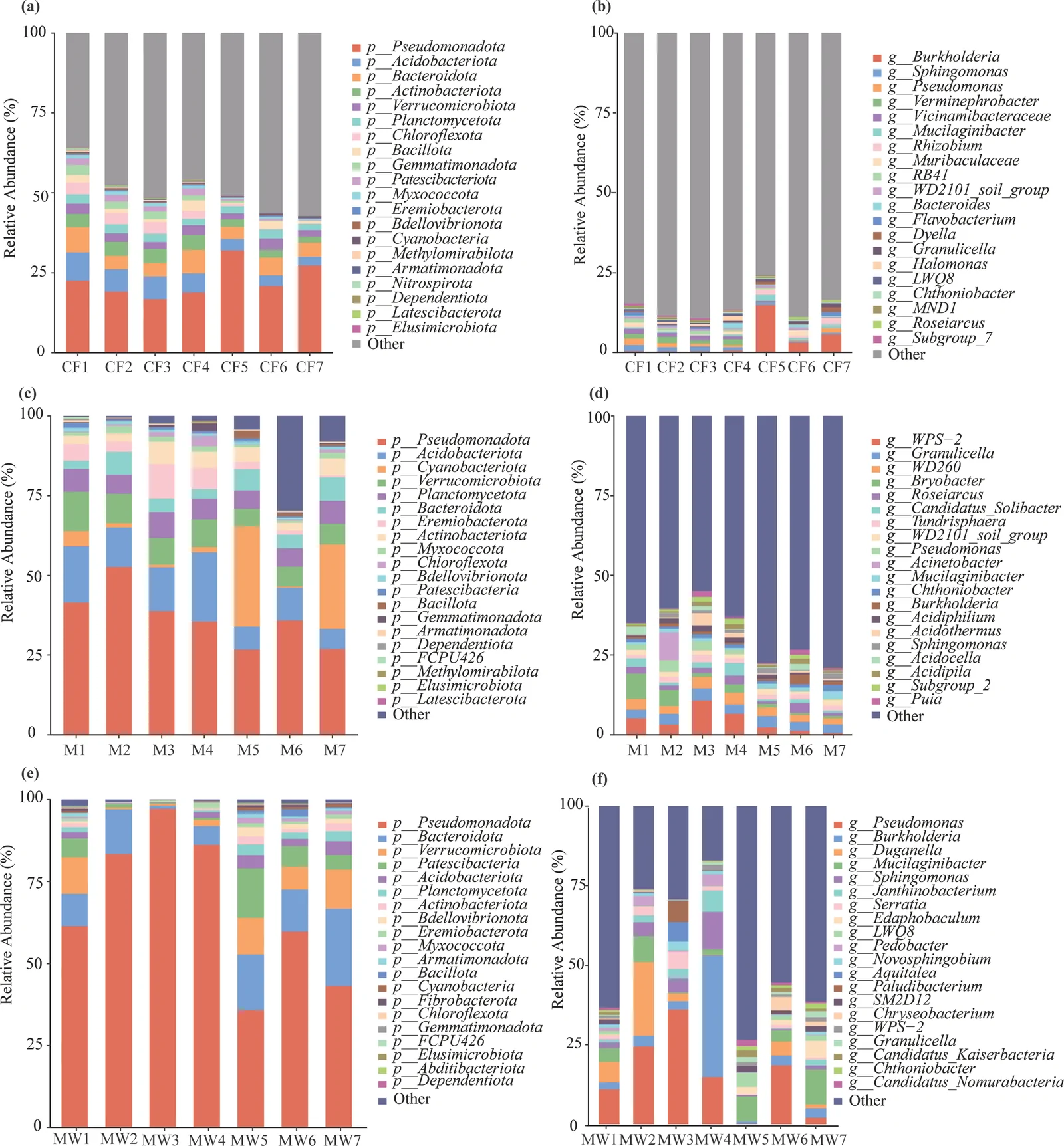
Relative abundance of the top 20 bacterial taxa at the phylum and genus levels. (**a**) Relative abundance of bacterial taxa at the phylum level in *C. fanjingshanensis*. (**b**) Relative abundance of bacterial taxa at the genus level in *C. fanjingshanensis*. (**c**) Relative abundance of bacterial taxa at the phylum level in moss layer. (**d**) Relative abundance of bacterial taxa at the genus level in moss layer. (**e**) Relative abundance of bacterial taxa at the phylum level in moss-water layer. (**f**) Relative abundance of bacterial taxa at the genus level in moss-water layer. CF, *C. fanjingshanensis*; M, different moss layers; and MW, different moss-water layers.

Statistical analyses were performed on the composition of bacterial communities at different taxonomic levels. The results showed that endophytic bacteria of *C. fanjingshanensis* belonged to 42 phyla, 122 classes, 334 orders, 535 families, and 1,202 genera; moss-layer bacteria belonged to 38 phyla, 108 classes, 276 orders, 440 families, and 830 genera; and moss-water-layer bacteria belonged to 37 phyla, 95 classes, 226 orders, 354 families, and 628 genera. At the phylum level, the dominant endophytic bacterial groups in CF were *Pseudomonadota* (16.69%–31.96%) and *Acidobacteriota* (2.69%–8.79%) (Fig. 5a). At the genus level, the main dominant genera were *Burkholderia* (0.49%–5.74%), *Sphingomonas* (0.38%–1.92%), and *Pseudomonas* (0.06%–2.06%), although their relative abundances varied among samples; for example, the abundance of *Burkholderia* in CF5 was notably higher than in other sampling sites (Fig. 5b). In moss-layer samples, dominant phyla were also *Pseudomonadota* (26.70%–52.62%) and *Acidobacteriota* (6.40%–21.67%) (Fig. 5c), while dominant genera differed among samples: M3 and M4 were dominated by WPS-2, whereas M1 and M5 were dominated by *Bryobacter* and *Granulicella*, respectively (Fig. 5d). In moss-water-layer samples, dominant phyla were *Pseudomonadota* (35.77%–97.18%) and *Bacteroidota* (0.943%–23.61%) (Fig. 5e). Dominant genera included *Pseudomonas* (0.08%–36.05%), *Burkholderia* (0.82%–38.33%), and *Duganella* (0.08%–23.19%), with considerable variation in relative abundance among different samples (Fig. 5f).

**Fig 6:**
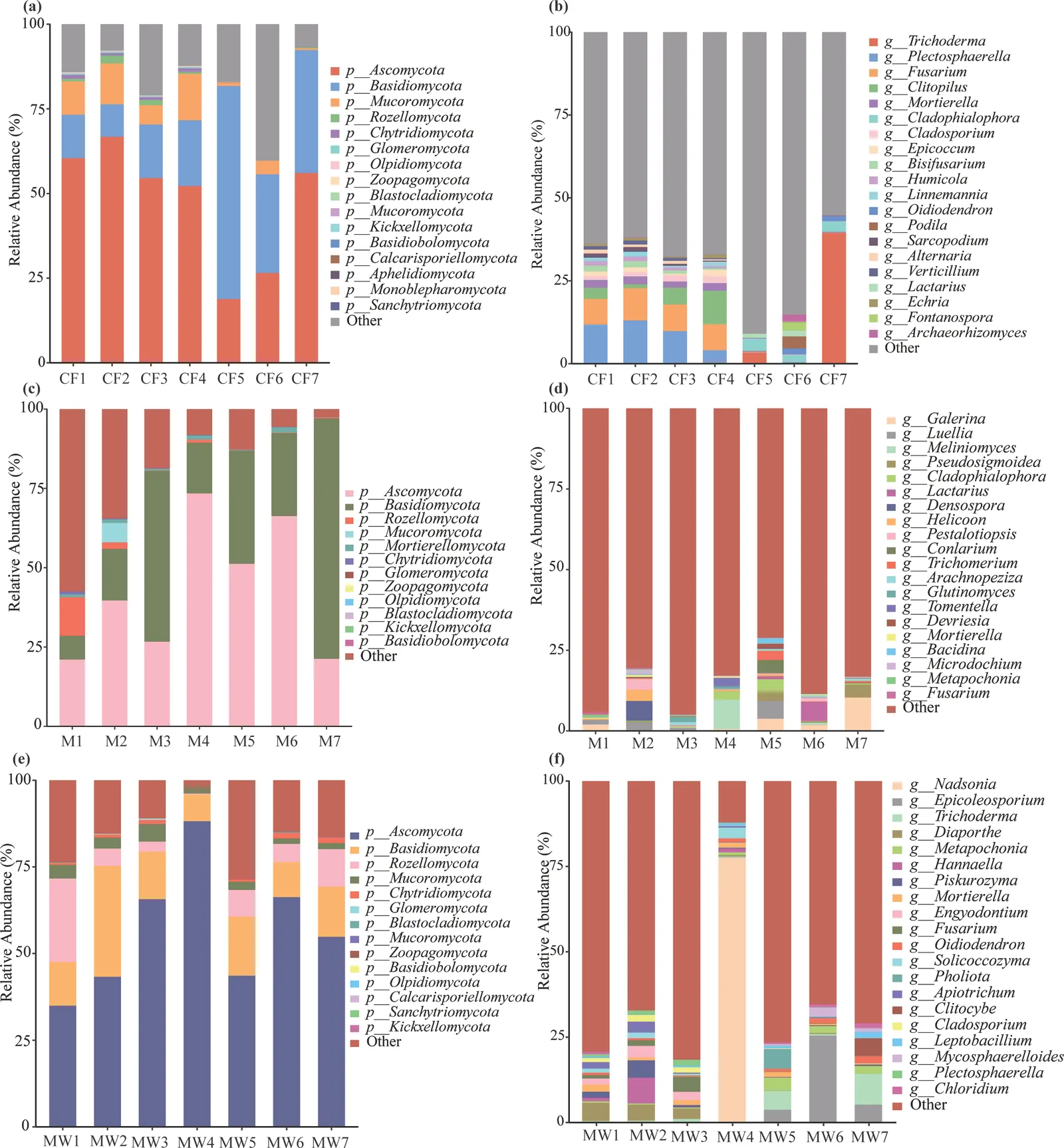
Relative abundance of the top 20 fungal species at the phylum and genus levels. (**a**) Relative abundance of fungi at the phylum level in *C. fanjingshanensis*. (**b**) Relative abundance of fungi at the genus level in *C. fanjingshanensis*. (**c**) Relative abundance of fungi at the phylum level in moss layer. (**d**) Relative abundance of fungi at the genus level in moss layer. (**e**) Relative abundance of fungi at the phylum level in moss-water layer. (**f**) Relative abundance of fungi at the genus level in moss-water layer. CF, *C. fanjingshanensis*; M, different moss layers; and MW, different moss-water layers.

Correspondingly, the endophytic fungal community of *C. fanjingshanensis* included 16 phyla, 57 classes, 152 orders, 359 families, and 948 genera; moss-layer fungi were distributed across 12 phyla, 43 classes, 124 orders, 305 families, and 715 genera; and moss-water-layer fungi comprised 14 phyla, 47 classes, 128 orders, 296 families, and 685 genera. At the phylum level, all three sample types were dominated by *Ascomycota* (18.84%–88.18%) and *Basidiomycota* (7.48%–75.75%) (Fig. 6a, c, and e). Similar to bacterial communities, fungal genera showed considerable variation among samples. Overall, the dominant endophytic fungal genera in CF were *Trichoderma* (0.06%–39.43%), *Plectosphaerella* (0.02%–12.89%), and *Fusarium* (0.09%–9.75%) (Fig. 6b); dominant genera in moss-layer samples were *Galerina* (0.01%–10.37%), *Luellia* (0%–5.54%), and *Meliniomyces* (0%–9.22%) (Fig. 6d); and in moss-water-layer samples, the dominant genera were *Nadsonia* (0%–77.47%), *Epicoleosporium* (0%–25.45%), and *Trichoderma* (0.25%–9.07%) (Fig. 6f).

In summary, the dominant bacterial phyla in CF endophytes and their habitats were *Pseudomonadota*, *Acidobacteriota*, and *Bacteroidota*, although the dominant bacterial genera varied markedly among samples. The main dominant fungal phyla were *Ascomycota* and *Basidiomycota*, and their dominant genera also exhibited considerable differences across sample types.

**Fig 7:**
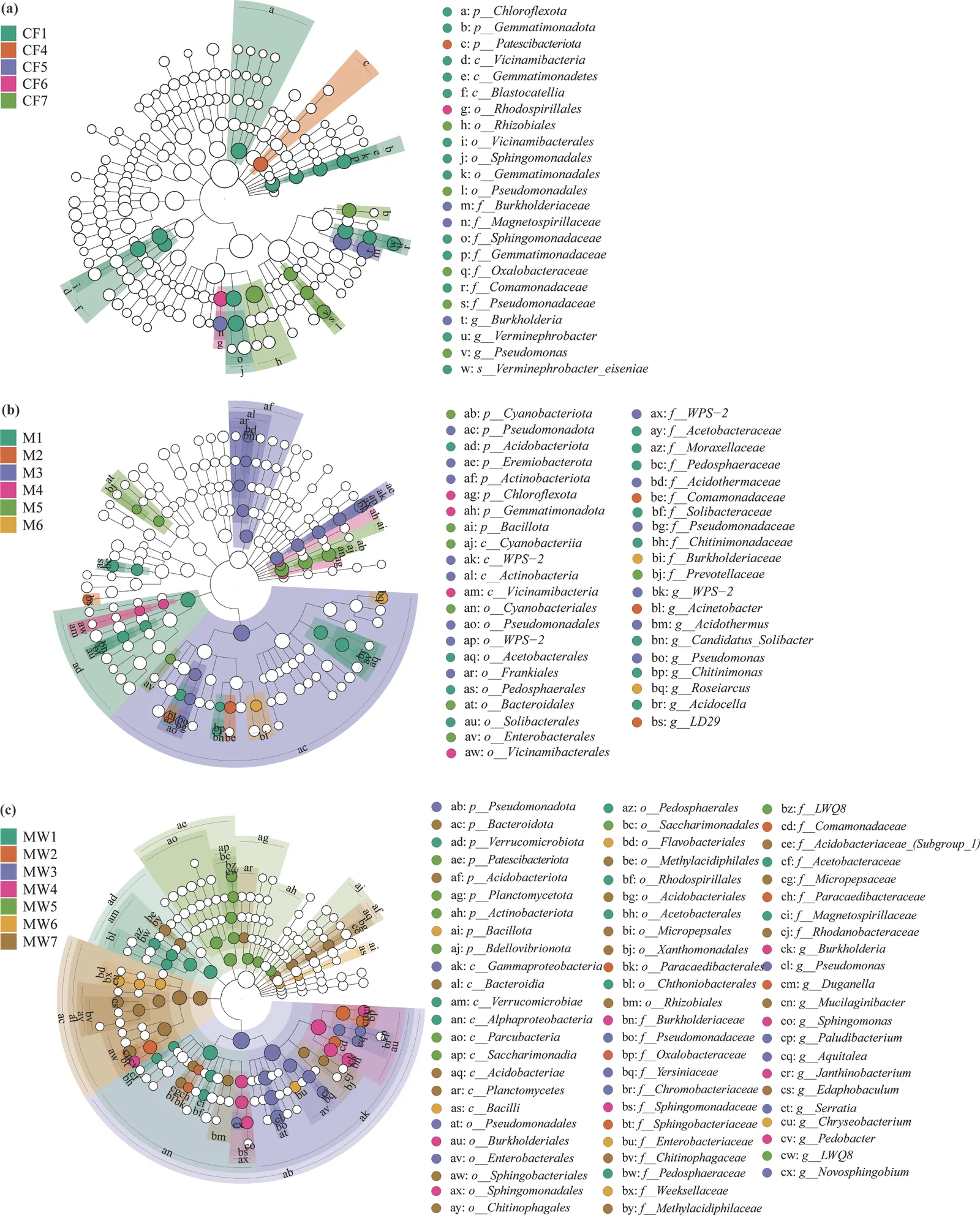
LEfSe cladograms of bacterial communities in different sample types. (**a**) *C. fanjingshanensis*; (**b**) moss layers; and (**c**) moss-water layers. Taxa from different sites are represented by colored circles. Only bacterial taxa with LDA significance >4 are included in the cladograms. Each cladogram consists of seven concentric circles representing, from the innermost to the outermost, domain, phylum, class, order, family, genus, and species. The size of each circle reflects the relative abundance of the corresponding taxon. CF, *C. fanjingshanensis*; M, different moss layers; and MW, different moss-water layers.

To investigate differences in microbial communities among sample groups, LEfSe was applied (LDA threshold >4.0 and *P* ≤ 0.05) to bacterial taxa in *C. fanjingshanensis*, moss layer, and moss-water-layer samples, considering only the top 200 abundant taxa. In the CF endophytic bacterial community, 60 differential taxa were identified, spanning five phyla, five classes, seven orders, seven families, and three genera. The CF1 sample contained the highest number of differential bacteria, with *Burkholderia*, *Verminephrobacter*, and *Pseudomonas* as dominant genera (Fig. 7a). In moss-layer samples, 42 differential bacterial taxa were detected, belonging to 9 phyla, 9 classes, 13 orders, 13 families, and 9 genera, with *WPS-2*, *Acinetobacter*, *Acidothermus*, and *Candidatus_Solibacter* as the dominant genera (Fig. 7b). In moss-water-layer samples, 71 differential bacterial taxa were identified, covering 9 phyla, 9 classes, 18 orders, 20 families, and 14 genera, with *Burkholderia*, *Pseudomonas*, *Duganella*, and *Mucilaginibacter* as the major dominant genera (Fig. 7c).

**Fig 8:**
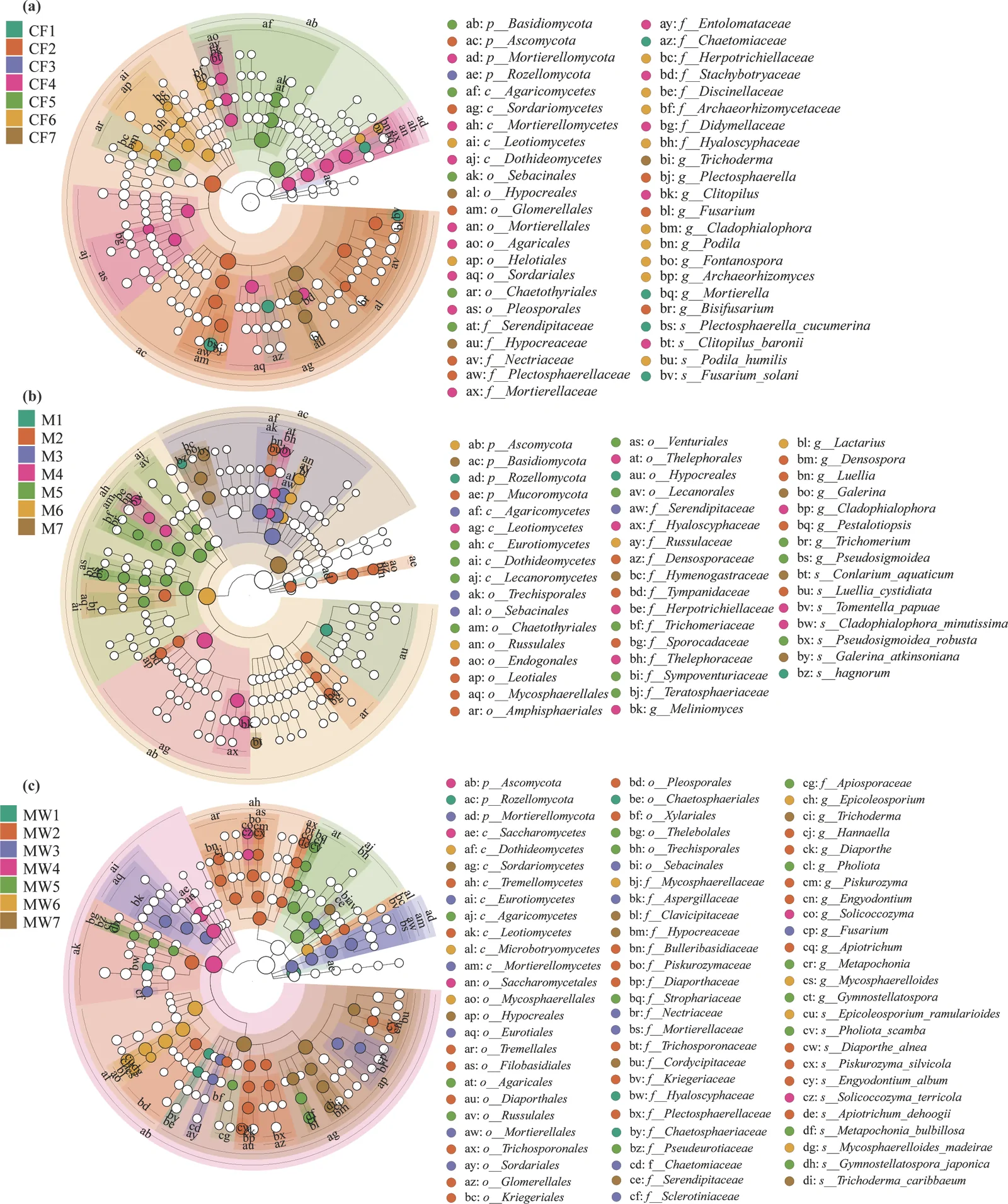
LEfSe cladograms of fungal communities in different sample types. (**a**) *C. fanjingshanensis*; (**b**) moss layers; and (**c**) moss-water layers. Only fungal taxa with LDA significance >4 are included in the cladograms. Each cladogram consists of seven concentric circles representing, from the innermost to the outermost, domain, phylum, class, order, family, genus, and species. The size of each circle reflects the relative abundance of the corresponding taxon. CF, *C. fanjingshanensis*; M, different moss layers; and MW, different moss-water layers.

Using the same thresholds, LEfSe analysis was also performed on fungal taxa (top 200 abundant taxa). In CF samples, 45 differential fungal taxa were identified, distributed across 4 phyla, 7 classes, 10 orders, 13 families, and 10 genera, with *Trichoderma*, *Plectosphaerella*, *Clitopilus,* and *Fusarium* as dominant genera (Fig. 8a). In moss-layer samples, 49 differential fungal taxa were detected, belonging to 4 phyla, 7 classes, 15 orders, 14 families, and 11 genera, with *Meliniomyces*, *Lactarius*, *Densospora*, and *Luellia* as dominant genera (Fig. 8b). In moss-water-layer samples, 81 differential fungal taxa were identified, covering 4 phyla, 9 classes, 22 orders, 21 families, and 15 genera, with *Epicoleosporium*, *Trichoderma*, *Hannaella*, and *Diaporthe* as the major dominant genera (Fig. 8c). These results demonstrate that distinct microhabitats shape significantly different microbial community structures and highlight, to some extent, the close associations between specific taxa and their corresponding ecological niches.

### Associations between endophytes and habitat microbiota and core microbe screening

**Fig 9:**
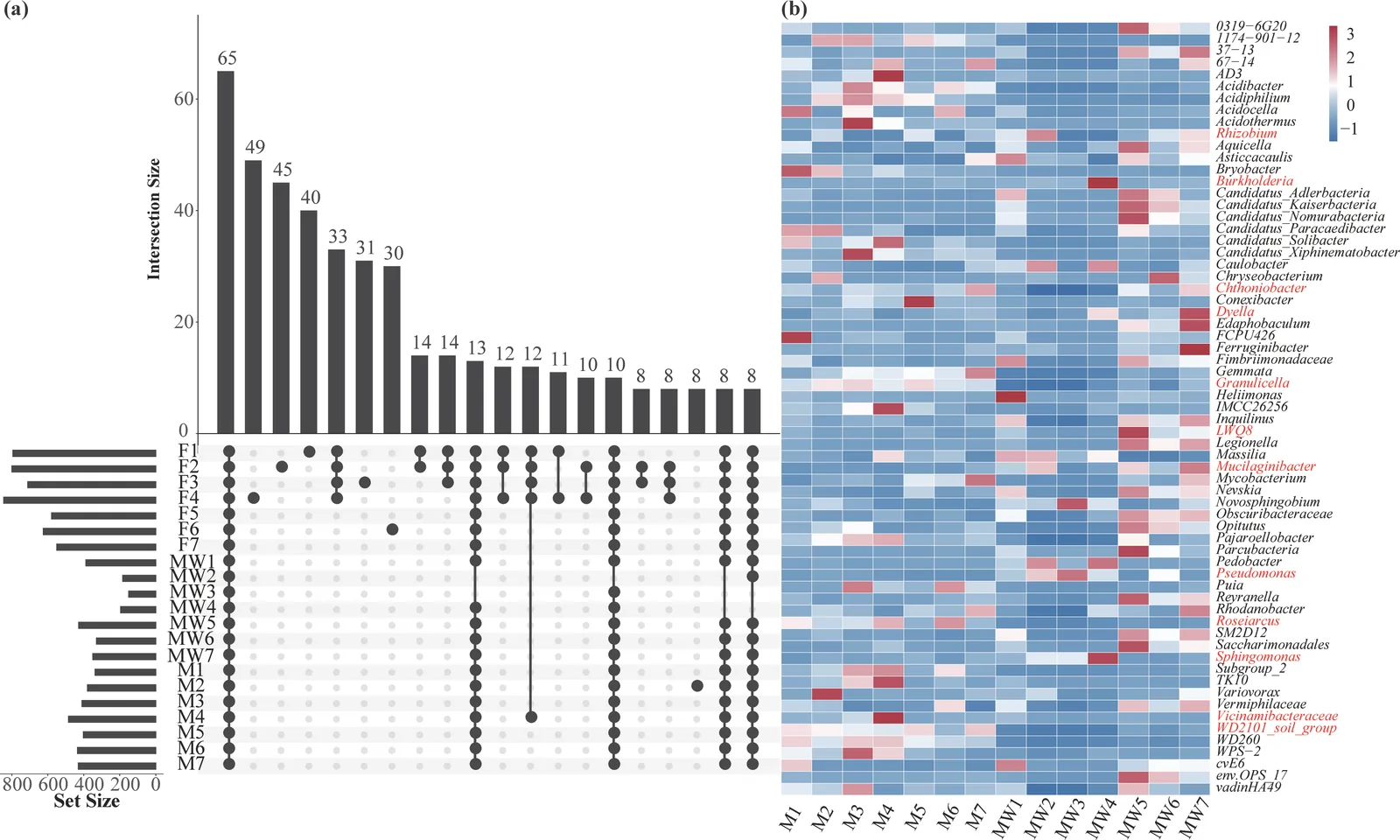
Shared core bacterial genera of *C. fanjingshanensis* and their relative abundance heatmap. (**a**) Shared core bacteria among CF, moss layers, and moss-water layers based on Venn analysis; (**b**) relative abundance heatmap of shared core bacteria in CF after *Z*-score normalization. Red indicates abundance above the genus average, and blue indicates abundance below the genus average. CF, *C. fanjingshanensis*; M, different moss layers; and MW, different moss-water layers.

To investigate the associations between *C. fanjingshanensis* endophytes and habitat microbiota, we used Venn analysis to identify the shared core bacterial genera among CF, M, and MW. The relative abundances of these shared core bacteria were *Z*-score standardized, and a heatmap was used to visualize their distribution differences across moss layers and moss-water layers. The results showed that 65 genera were shared among the three sample types (Fig. 9a), which were defined as the shared core endophytic bacteria of *C. fanjingshanensis*. Their distribution differences in moss layers and moss-water layers are shown in Fig. 9b. Based on this, we further selected the top 20 most abundant shared core endophytic bacteria in *C. fanjingshanensis* (marked in red), which mainly included *Rhizobium*, *Burkholderia*, *Pseudomonas*, and *Sphingomonas* (12 genera in total), predominantly distributed in moss-water layers.

**Fig 10:**
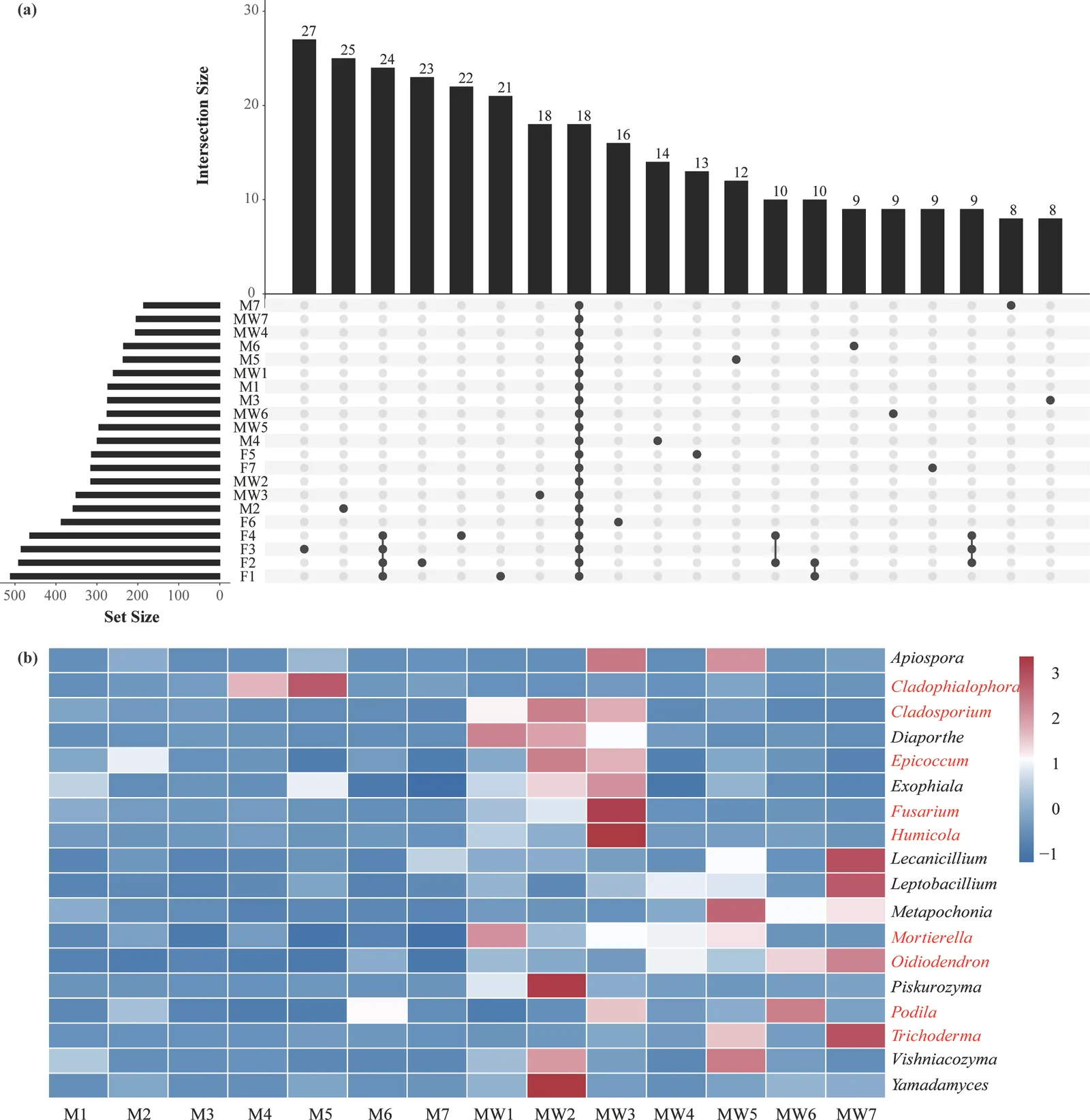
Shared core fungal genera of *C. fanjingshanensis* and their relative abundance heatmap. (**a**) Shared core fungi among CF, moss layers, and moss-water layers based on Venn analysis; (**b**) relative abundance heatmap of shared core fungi in CF after *Z*-score normalization. Red indicates abundance above the genus average, and blue indicates abundance below the genus average. CF, *C. fanjingshanensis*; M, different moss layers; and MW, different moss-water layers.

Similarly, 18 shared core fungal genera were identified among the three sample types (Fig. 10a), and their distributions in moss layers and moss-water layers are shown in Fig. 10b. Further screening of the top 20 most abundant shared core endophytic fungi in *C. fanjingshanensis* (marked in red) identified 9 genera, including *Trichoderma*, *Mortierella*, *Oidiodendron*, and *Epicoccum*, which were also mainly concentrated in moss-water layers.

### Microbial co-occurrence network and gene function prediction analysis

**Fig 11:**
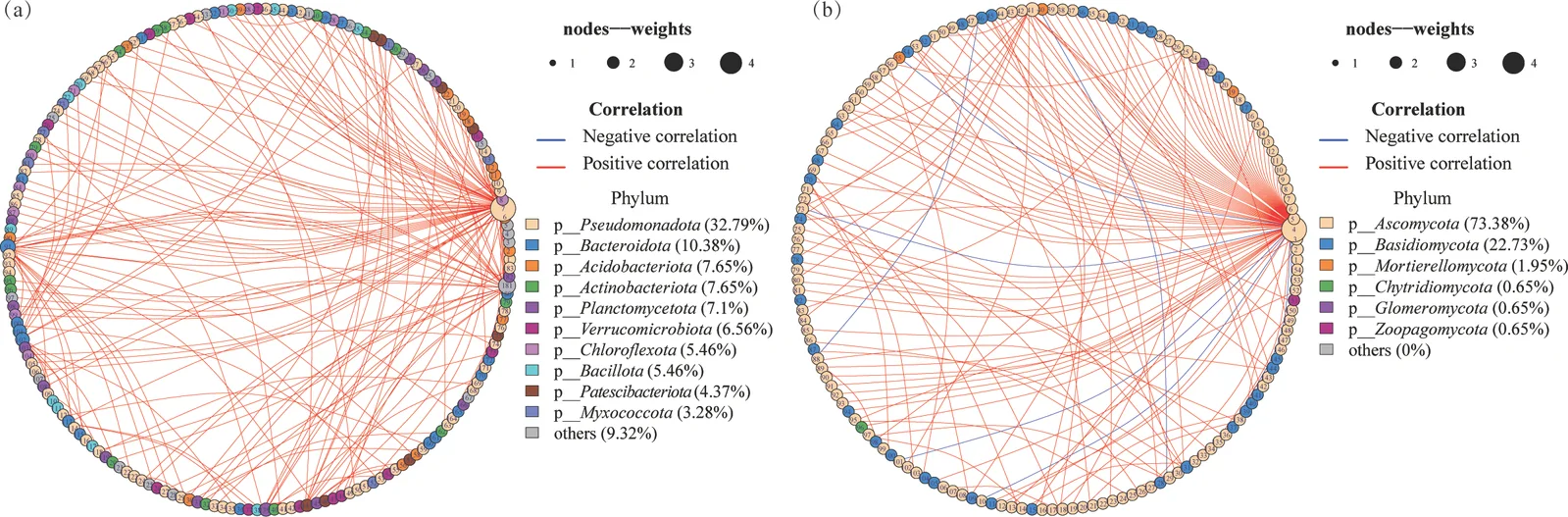
Co-occurrence network of bacterial and fungal genera in the *C. fanjingshanensis*–moss substrate symbiotic system. (**a**) Bacterial network; (**b**) fungal network. Each node corresponds to a bacterial or fungal genus, while the edges denote significant correlations (Spearman’s *r* > 0.6, *P* < 0.05) between them. Red edges represent positive correlations, whereas blue edges indicate negative ones.

To elucidate the potential interactions among the microbiota within the *C. fanjingshanensis*–moss substrate symbiotic system, the top 200 bacterial and fungal genera with the highest relative abundances from mixed samples of *C. fanjingshanensis*, moss layers, and moss-water layers were selected for Spearman rank correlation analysis. Genera with correlation coefficients |*r*| > 0.6 and *P* < 0.05 were used to construct co-occurrence networks, and the corresponding network topological properties were calculated. The results (Fig. 11a and b; Table S2) revealed a predominance of positive correlations, with positive edge proportions of 1.00 for bacteria and 0.942 for fungi. This indicates that synergistic and mutualistic interactions dominate microbial associations, which may facilitate nutrient sharing and recycling. Such cooperative relationships likely enhance nutrient supply to *C. fanjingshanensis* while improving the efficiency of resource utilization within the moss substrate, thereby maintaining metabolic balance in the symbiotic system. Both bacterial (modularity = 0.783) and fungal (modularity = 0.586) networks exhibited modular organizational structures, suggesting function-based clustering that enables microorganisms with similar ecological roles to collaborate efficiently within modules. Moreover, relatively short average path lengths (3.511 for bacteria and 3.564 for fungi) allow for rapid information transfer within the community, enabling the symbiotic system to promptly respond to environmental fluctuations and jointly enhance the adaptability and ecological stability of the *C. fanjingshanensis*–moss substrate complex in alpine environments.

According to the node characteristics in the co-occurrence networks (Fig. S1a and b; Table S3), key microbial taxa (module hubs) were identified, including seven bacterial and two fungal nodes. These comprised *Vicinamibacteraceae* of the phylum *Acidobacteriota*, *Verminephrobacter* of *Pseudomonadota*, *Mucilaginibacter* and *Muribaculaceae* of *Bacteroidota*, *WD2101_soil_group* of *Planctomycetota*, *LWQ8* of *Patescibacteriota*, *WPS-2* of *Eremiobacterota,* and the fungal genera *Plectosphaerella* and *Epicoleosporium* of *Ascomycota*. A further comparison between the shared core bacteria and the key taxa revealed five overlapping bacterial groups: *Vicinamibacteraceae*, *Mucilaginibacte*r, *WD2101_soil_group*, *LWQ8*, and *WPS-2*. These were defined as the key shared core bacterial taxa, as indicated in Table S3 and highlighted in red.

**Fig 12:**
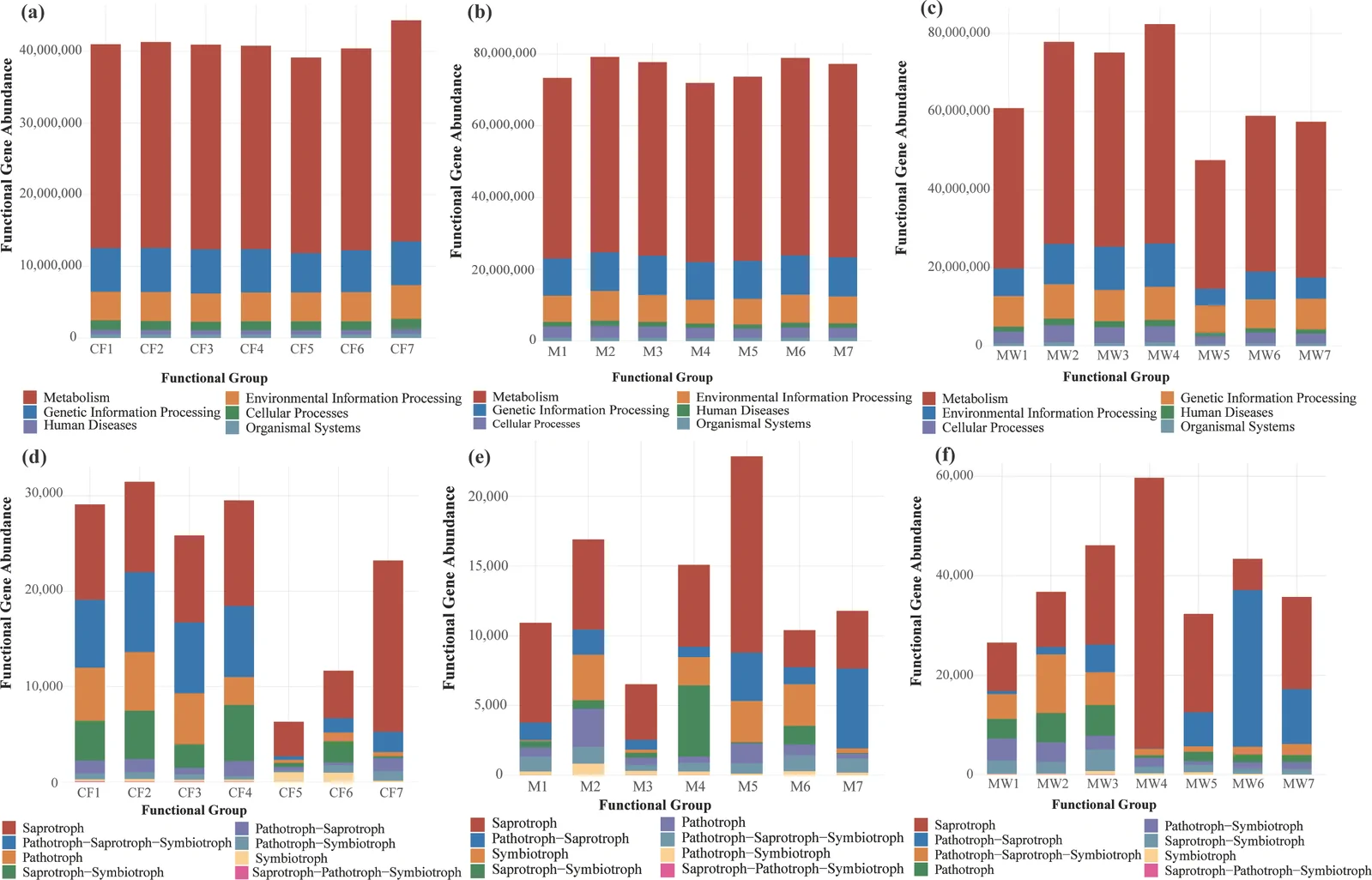
Abundance of microbial functional genes. (**a**) Functional gene abundance of endophytic bacteria; (**b**) functional gene abundance of bacteria in the moss layer; (**c**) functional gene abundance of bacteria in the moss-water layer; (**d**) functional gene abundance of endophytic fungi; (**e**) functional gene abundance of fungi in the moss layer; (**f**) functional gene abundance of fungi in the moss-water layer. CF, *C. fanjingshanensis*; M, different moss layers; and MW, different moss-water layers.

Bacterial and fungal gene functions were predicted using PICRUSt2 and FUNGuild, respectively, and functional gene abundance bar plots were generated for different sample types (Fig. 12). In general, bacterial functional gene abundances remained relatively stable among sampling sites within groups, while fungal functional genes demonstrated more significant variation. Specifically, the relative abundances of bacterial functional genes in endophytic and moss-layer samples exhibited only minor variations among sampling sites, whereas those in moss water samples fluctuated considerably (Fig. 12a through c). This suggests that the bacterial functional differentiation between the endophytic and moss-layer environments was limited, while the more dynamic nutrient and moisture conditions in moss-water habitats may have driven the divergence in bacterial gene expression. Across all sample types, bacterial genes were consistently enriched in categories related to Metabolism, Environmental Information Processing, and Genetic Information Processing. This indicates their capacity to sense environmental signals, regulate metabolic processes, and maintain genetic stability, thereby playing a central role in nutrient absorption, stress resistance, and maintaining host–microbe symbiosis.

In contrast, the relative abundances of fungal functional genes varied markedly among sampling sites within each group, with particularly pronounced differences observed in moss-water-layer samples (Fig. 12d through f). Overall, saprotroph-associated genes dominated the samples from both *C. fanjingshanensis* and the moss layer, whereas the abundance of saprotroph and pathotroph-saprotroph trophic genes varied significantly across the moss-water-layer samples. These patterns suggest that environmental heterogeneity among sample types exerts distinct selective pressures on fungal trophic strategies. Endophytic fungi primarily adopt saprotrophic metabolism to support the host, moss-layer fungi balance organic matter decomposition with symbiotic interactions, and moss-water-layer fungi employ diverse trophic modes to utilize fluctuating nutrients and facilitate nutrient transfer across habitats.

In summary, we propose that the endophytic microbiota of *C. fanjingshanensis* supports the host’s fundamental adaptability through bacterial metabolic activity and fungal saprotrophic–symbiotic strategies. In the moss layer, bacterial nutrient activation and fungal symbiotic interactions help improve the microhabitat. In the moss-water layer, microorganisms respond metabolically to dynamic microenvironments, while diverse fungal trophic modes facilitate nutrient transfer across habitats. Collectively, these processes establish a functional network integrating material cycling, nutrient interaction, and stress response, thereby enhancing the resilience of the *C. fanjingshanensis*–moss symbiotic system to extreme alpine conditions characterized by low temperature and nutrient limitation.

## DISCUSSION

During the process of *ex situ* plant conservation, endophytic and habitat-associated microorganisms together constitute the host plant’s microecosystem, and their interactions play a crucial role in influencing the plant’s post-transplantation adaptability and survival rate (46, 47). Endophytic microbes colonize plant tissues and enhance host stress tolerance, promote nutrient absorption, and induce systemic resistance (48–50), while habitat microbes indirectly support plant health through nutrient cycling, pathogen suppression, and microenvironmental regulation (51–53). The success of *ex situ* conservation depends not only on the physical transplantation of plants but also on the preservation and introduction of native microbial communities to mitigate the loss of microbial functions caused by habitat changes. Moreover, microbial-assisted restoration strategies can further enhance the ecological establishment and long-term persistence of plants in new environments.

In this study, we systematically analyzed the overall community characteristics of endophytic and habitat microorganisms associated with *C. fanjingshanensis*, as well as their interrelationships. We further explored the unique patterns of plant–microbe interactions and their synergistic adaptive strategies to environmental stress within the “sky island” habitat. These findings contribute to a deeper understanding of the co-evolutionary mechanisms between plants and microorganisms under extreme environments and provide a scientific basis for microbial-assisted conservation of plant species with extremely small populations.

### Differentiation of endophytic microbial communities and functional adaptation

ASV clustering analysis revealed that the number of unique endophytic bacterial and fungal ASVs was significantly higher than that in moss layer and moss-water layer, while the three sample types shared only three bacterial and fungal ASVs. This indicates a strong selective effect of the *C. fanjingshanensis* endophytic environment on microbial colonization. Such specificity likely arises from the unique microenvironment within plant tissues, which allows only a limited number of highly adaptable microorganisms to establish. Notably, *Pseudomonadota*, *Acidobacteriota*, *Ascomycota*, and *Basidiomycota* were significantly enriched in *C. fanjingshanensis. Pseudomonadota* often possess nitrogen-fixing or phosphorus-solubilizing capabilities (54–57), while *Ascomycota* fungi are generally tolerant to extreme environments and highly efficient in organic matter decomposition (58, 59). These microbial groups may play critical roles in enabling *C. fanjingshanensis* to cope with nutrient-limited and low-temperature conditions characteristic of alpine habitats.

Alpha diversity analysis showed that endophytic bacterial communities exhibited lower Shannon, Chao1, and Sobs indices, but higher dominance, indicating that the community structure is dominated by specific keystone taxa. This pattern of low diversity and high dominance may be an adaptive strategy in extreme environments, which can reduce interspecific competition and enhance functional cooperation to improve resource utilization efficiency. In contrast, moss-water-layer fungi showed the highest Shannon index and species richness, likely due to the openness of their habitat and water-mediated dispersal, supporting ongoing microbial exchange with the surrounding environment. Moss layers, as the substrate directly associated with *C. fanjingshanensis*, may serve as a “microbial reservoir” prior to endophytic colonization, providing a potential source of functional microbes for the plant.

PCoA and ANOSIM tests further confirmed that microbial community structures significantly differed among *C. fanjingshanensis*, moss layers, and moss-water layers, indicating that microenvironmental heterogeneity is an important driver of microbial niche differentiation. The endophytic environment, moss layers, and moss-water layers selectively maintain microbial groups adapted to their specific physicochemical conditions, reflecting a tight interaction and co-adaptation among microbes, plants, and their environment. Therefore, for the effective conservation of *C. fanjingshanensis*, it is essential to not only maintain its population size but also prioritize the integrity of its microhabitat, thereby transitioning from single-species conservation to a holistic “species-microbe-habitat” protection framework.

### Assembly of endophytic microbial communities and environmental adaptation mechanisms

The shared core endophytic bacteria and fungi of *C. fanjingshanensis* were mainly derived from moss-water layers, indicating that moss-water layers in alpine ecosystems may simultaneously function as both microbial “sources” and “sinks.” Moss-water layers may adsorb airborne microorganisms to form a regional species pool (60) and potentially transport them into plant tissues through open structures such as stomata, lenticels, or wounds (21, 61), serving as a crucial pathway for endophytic colonization. This process may be regulated by a dispersal–colonization trade-off, in which highly mobile taxa in moss-water layers exhibit stronger dispersal abilities, while selective pressures within the host may retain taxa capable of both dispersal and stable colonization. Previous studies also suggested that negative correlations dominate bacterial networks in moss-water layers, reflecting significant resource competition and possibly selecting for taxa with high dispersal efficiency (62). In addition, the low temperatures of high-altitude environments may further select for cold-tolerant microbial groups in moss-water layers, which may then establish as core endophytes, potentially enhancing the host’s tolerance to alpine stresses.

The shared core microbiota identified in this study consists primarily of beneficial taxa. *Burkholderia* may synthesize antimicrobial secondary metabolites and provide both biocontrol and growth-promoting functions (63). *Sphingomonas* may degrade soil pollutants and play important roles in environmental remediation (64, 65). *Rhizobium* may promote the growth of non-leguminous host plants through associative nitrogen fixation, nutrient solubilization, and the production of phytohormones (66–70). *Trichoderma* may directly antagonize pathogenic fungi, playing a key role in biological control (71–73). *Mortierella* may activate soil nutrients, promoting carbon, nitrogen, and phosphorus cycling and rapid nutrient release (74).

Taken together, our findings suggest that the moss-water layer serves as a crucial microbial reservoir for the shared core microbiome of *C. fanjingshanensis*, indicating that the assembly of the endophytic community is likely shaped by the combined effects of environmental dispersal and host filtering. Furthermore, the shared core microbiome is enriched with taxa associated with nutrient mobilization and absorption, as well as resistance to pathogenic fungal infections, which may assist the host in adapting to high-alpine nutrient limitation and low-temperature stress. However, since our current findings are based solely on amplicon sequencing, functional predictions, and literature references, future empirical studies—such as microbial inoculation trials and meta-transcriptomics—are required to validate these potential physiological benefits. Nevertheless, the exploration of these correlative patterns highlights the importance of microhabitat integrity, suggesting that conservation strategies for rare alpine plants should consider preserving not only the target species but also their surrounding microbial reservoirs. In future *ex situ* conservation practices, microbial community transplantation techniques could be applied to simulate and inoculate native moss-water-layer microbes, thereby rebuilding symbiotic relationships and enhancing the survival rates of translocated populations.

### Microbial co-occurrence networks and their contribution to adaptive strategies

Co-occurrence networks are widely used to reveal interactions among microbial taxa, providing a visual representation of relationships within microbial communities and reflecting their survival strategies in specific habitats (75). The microbial network of *C. fanjingshanensis* and its moss substrate exhibits strong positive interactions, suggesting that endophytes and habitat-associated microbes may reach a relatively mature level of interaction. Microbial interactions are predominantly cooperative and mutually beneficial, with functional complementarity enhancing the community’s collective ability to cope with adverse environmental conditions (76–78). The relatively short average path length in the network may facilitate rapid transmission of information and materials between microbial taxa, allowing timely responses to habitat fluctuations, which is particularly important for adaptation to the unstable “sky island” environment.

Identification of key core microbes further highlights critical functional nodes. Microbes such as *Vicinamibacteraceae*, *Mucilaginibacter*, and *WD2101_soil_group* are not only highly associated with host adaptation but also serve as essential modules maintaining network integrity. The loss of these taxa could disrupt network connectivity and reduce the host’s adaptive capacity, indicating that these microbes may represent critical targets for future conservation efforts.

### Conclusion

The endophytic environment of *C. fanjingshanensis* exerts a strong filtering effect on habitat microorganisms; the number of unique endophytes is significantly higher than in the moss layer and the moss-water layer. Furthermore, the dominance of endophytic bacteria is high, and its community structure is primarily dominated by specific dominant bacteria. The microbial co-occurrence network of the “*C. fanjingshanensis*–moss substrate” exhibits extremely strong positive interactions and a modular structure, which facilitates synergistic resistance against adverse environments; the core microorganisms of *C. fanjingshanensis* primarily originate from the moss-water layer, enriching numerous taxa related to nutrient acquisition and stress resistance. The functional gene abundances of endophytic, moss layer, and moss-water-layer bacteria vary relatively smoothly among intra-group sampling sites, whereas the fungal functional gene abundances of the three sample types exhibit obvious variations among intra-group sampling sites. Notably, the saprotroph and pathotroph-saprotroph trophic genes differ significantly in the moss-water layer, indicating that the selection pressure of environmental heterogeneity on fungal trophic strategies is more pronounced.

This study proposes a holistic conservation scheme that relies on the native moss-water layer to conduct microbial community transplantation, directionally multiply key core microbiota, protect moss microhabitat integrity, and establish a long-term microbial monitoring system, providing a theoretical reference for the microbiome-assisted conservation of extremely narrowly distributed alpine orchid species.

## Data Availability

High-throughput sequencing data were deposited in the NCBI database (https://www.ncbi.nlm.nih.gov/). Bacterial data were stored under project numbers PRJNA1267579 and PRJNA1267769; fungal data were stored under project numbers PRJNA1267777 and PRJNA1267781. The scripts and feature-ASV-table-taxonomy table have been uploaded to the public code hosting platform GitHub (https://github.com/zouhuakai/Article2-Script). All the data analysis results obtained in this study have been included in the manuscript, along with its accompanying figures and tables. Moreover, the plant specimens of *C. fanjingshanensis* were stored in Guizhou Provincial Botanical Garden, Guizhou, China.

## SUPPLEMENTAL MATERIAL

Fig. S1; Tables S1 to S3 spectrum.03719-25-s0001.docx Node parameters and key taxa of microbial network analysis; Dominant moss species at different sampling sites.

ASM does not own the copyrights to Supplemental Material that may be linked to, or accessed through, an article. The authors have granted ASM a non-exclusive, world-wide license to publish the Supplemental Material files. Please contact the corresponding author directly for reuse.
