# Supplementary material for: Microbial insights into the survival adaptation of the endemic orchid Corybas fanjingshanensis in a “sky island” habitat
Source: Microbiol Spectr. 2026 Oct 6;14(11):e03719-25. doi: 10.1128/spectrum.03719-25 (PMC13729222; doi:10.1128/spectrum.03719-25)
Supplement: Supplementary file 1 [file spectrum.03719-25-s0001.docx]

**FIGURE S1 Zi-Pi analyst of** C. fanjingshanensis**–moss substrate symbiont.** (a) Zi-Pi distribution of bacteria; (b) Zi-Pi distribution of fungus.

**Table. S1** Seven major types of mosses that coexist with *C. fanjingshanensis*

| Number | Dominant moss types | Family |
| --- | --- | --- |
| 1 | *Sphagnum junghuhnianum* | [Sphagnaceae](https://www.iplant.cn/info/Sphagnaceae) |
| 2 | *Hypnum cupressiforme* | [Hypnaceae](https://www.iplant.cn/info/Hypnaceae) |
| 3 | *Sinkea flammea* | [Meteoriaceae](https://www.iplant.cn/info/Meteoriaceae) |
| 4 | *Dicranodontium filifolium* | [Dicranaceae](https://www.iplant.cn/info/Dicranaceae) |
| 5 | *Plagiochila semidecurrens* | [Plagiochilaceae](https://www.iplant.cn/info/Plagiochilaceae) |
| 6 | *Wijkia deflexifolia* | [Sematophyllaceae](https://www.iplant.cn/info/Sematophyllaceae) |
| 7 | *Dicranum drummondii* | [Dicranaceae](https://www.iplant.cn/info/Dicranaceae) |

**Table S2** Microbial network attribute parameters in *C. fanjingshanensis*–moss substrate symbiont.

|  | Bacteria | Fungus |
| --- | --- | --- |
| Nodes | 183 | 154 |
| Edges | 183 | 154 |
| Average degree | 2 | 2 |
| Average path distance | 3.511 | 3.564 |
| Postive Edges | 183 | 145 |
| Negative Edges | 0 | 9 |
| Density | 0.011 | 0.013 |
| Modularity | 0.783 | 0.586 |
| Diameter | 27.056 | 33.234 |
| Positive edges ratio | 1.00 | 0.942 |

**Table S3** Distribution of key microbial groups in *C. fanjingshanensis*–moss substrate symbiont. Red indicates the key shared core bacteria.

| [Different position](javascript:;) | Generic level | [Phylum level](javascript:;) | [Type](javascript:;) | [Abundance](javascript:;) |
| --- | --- | --- | --- | --- |
| Bacteria | *Vicinamibacteraceae* | *Acidobacteriota* | Module hubs | 0.006 |
|  | *Verminephrobacter* | *Pseudomonadota* | Module hubs | 0.007 |
|  | *Mucilaginibacter* | *Bacteroidota* | Module hubs | 0.016 |
|  | *Muribaculaceae* | *Bacteroidota* | Module hubs | 0.005 |
|  | *WD2101_soil_group* | *Planctomycetota* | Module hubs | 0.007 |
|  | *LWQ8* | *Patescibacteriota* | Module hubs | 0.005 |
|  | *WPS-2* | *Eremiobacterota* | Module hubs | 0.011 |
| Fungus | *Plectosphaerella* | *Ascomycota* | Module hubs | 0.037 |
|  | *Epicoleosporium* | *Ascomycota* | Module hubs | 0.008 |
